# Internal Variability-Generated Uncertainty in East Asian Climate Projections Estimated with 40 CCSM3 Ensembles

**DOI:** 10.1371/journal.pone.0149968

**Published:** 2016-03-01

**Authors:** Shuai-Lei Yao, Jing-Jia Luo, Gang Huang

**Affiliations:** 1 State Key Laboratory of Numerical Modeling for Atmospheric Sciences and Geophysical Fluid Dynamics, Institute of Atmospheric Physics, Chinese Academy of Sciences, Beijing, 100029, China; 2 University of Chinese Academy of Sciences, Beijing, 100049, China; 3 Bureau of Meteorology, Melbourne, VIC, 3008, Australia; 4 Joint Center for Global Change Studies, Beijing, 100875, China; Universidade de Vigo, SPAIN

## Abstract

Regional climate projections are challenging because of large uncertainty particularly stemming from unpredictable, internal variability of the climate system. Here, we examine the internal variability-induced uncertainty in precipitation and surface air temperature (SAT) trends during 2005–2055 over East Asia based on 40 member ensemble projections of the Community Climate System Model Version 3 (CCSM3). The model ensembles are generated from a suite of different atmospheric initial conditions using the same SRES A1B greenhouse gas scenario. We find that projected precipitation trends are subject to considerably larger internal uncertainty and hence have lower confidence, compared to the projected SAT trends in both the boreal winter and summer. Projected SAT trends in winter have relatively higher uncertainty than those in summer. Besides, the lower-level atmospheric circulation has larger uncertainty than that in the mid-level. Based on k-means cluster analysis, we demonstrate that a substantial portion of internally-induced precipitation and SAT trends arises from internal large-scale atmospheric circulation variability. These results highlight the importance of internal climate variability in affecting regional climate projections on multi-decadal timescales.

## Introduction

Changes in regional climate are particularly relevant for effective decision-making on how to manage adaptation and mitigation, and how to cope with potential losses and damages at the regional scale in a future warmer climate [1]. However, projections of regional climate change are characterized by considerable uncertainty, which has emerged as a pressing challenge in climate science [2]. Uncertainty in regional climate projections mostly comes from three distinct sources [3–7]. The first source is model-response uncertainty: each global climate model may produce different future responses to the same prescribed external radiative forcing due to different physics, dynamical cores and resolutions, as well as model biases [8]. The second is emission-scenario uncertainty, which arises from the uncertainties in future trajectory of external radiative forcing, including time-dependent emissions of greenhouse gases and particles, precursor pollutant compounds, and land use/cover changes [9]. The third is natural variability of the climate system, which mainly stems from processes intrinsic to atmosphere, land, ocean and cryosphere, and their coupled interactions [10]. The first two types of uncertainties may be potentially reduced as climate models are improved and emissions scenarios become more accurate. However, to a large extent, the third type of uncertainty is mostly unable to be reduced owing to the inherently unpredictable and spontaneous nature of internal variability of the climate system.

The uncertainty in past and projected precipitation and surface air temperature (SAT) trends that is attributable to internal variability has been discussed extensively [11, 12], but the emphases in many previous studies have mostly been paid on examining the statistical significance of projected climate trends rather than on understanding physical mechanisms of internally-generated variability that influences future regional climate trends. Regional climate projections are susceptible to influences from internal variability of the climate system for time periods of several decades and even longer. Numerous studies have demonstrated that much of regional-scale precipitation and SAT trends on multi-decadal timescales is mediated by variations in the large-scale atmospheric circulation [6, 13]. A recent study based on large ensembles of climate change simulations using two comprehensive climate models has shown that North American climate trends during 2010–2060 are subject to high uncertainty primarily arising from internal large-scale atmospheric circulation variability [14]. Whether similar uncertainties in future regional climate projections are present in East Asia needs to be assessed. In East Asia where a large percentage of the world’s populations are vulnerable to climate fluctuations and changes [15], it is of vital consequences to investigate the contribution of internal variability to future East Asian climate projections on multi-decadal timescales.

This purpose of this study is to employ large ensemble simulations to examine regional-scale uncertainty in future East Asian climate projections. We first analyze the relative importance of the externally-forced and internally-generated components in projected future East Asian climate trends. We then examine the impact of the internal climate variability upon future climate changes for East Asia, and assess the internal variability-induced uncertainties on multi-decadal timescales.

## Materials and Methods

The Community Climate System Model Version 3 (CCSM3) is a comprehensive coupled atmosphere-ocean-sea ice-land general circulation model, which consists of Community Atmospheric Model Version 3 at 2.8° horizontal resolution (T42 spectral truncation) and 26 levels in the vertical, Parallel Ocean Program at 1° horizontal resolution with increased resolution to 0.32° at the equator and 40 levels in the vertical, Community Sea Ice Model Version 5 with plastic-elastic-viscous dynamics, and Community Land Model described in Collins, Bitz [16], to which readers are referred for details.

The 40 member ensembles of climate change simulations using the CCSM3 for the period 2000–2060 are conducted. Each of the 40 ensemble members is driven by an identical time-varying radiative forcing: the SRES A1B scenario-based greenhouse gas and stratospheric ozone as well as sulfate aerosol and black-carbon changes [17]. In the 40 realizations, the initial states in the ocean, sea ice and land are identical but a suite of different atmospheric initial conditions are taken from different days between December 1999 and February 2000 from the twentieth-century CCSM3 simulation. Note that perturbing the ocean initial conditions may further increase the uncertainty of climate projections, but this is beyond the scope of this study. Readers are referred to [6] for the details of the ensemble scheme. If assuming the model and external forcing scenario are perfect, the trajectory of each single member of the ensembles would give one plausible outcome of climate change in the presence of internal variability. Spread of climate trends among the different ensemble members represents the irreducible uncertainty inherent in regional climate projections. The externally-forced climate change can be isolated from the internal variability of the climate system based on the large ensemble simulations. By assuming the model is perfect, the 40 member ensemble mean represents the externally-forced climate change signal and thus the differences of the individual realizations from the 40 member ensemble mean represent the internal variability of the simulated climate system. In this study, we define that the externally-forced climate change is statistically robust if more than 90% of the 40 members produce the same sign of trend at each model grid.

**Fig 1:**
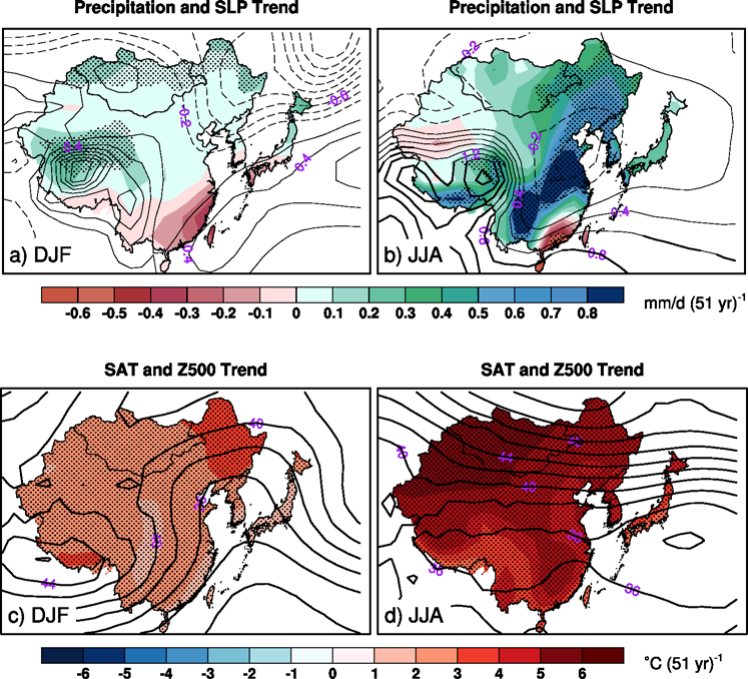
The boreal winter (DJF) and summer (JJA) precipitation and sea level pressure, and surface air temperature and 500hPa geopotential height trends during 2005–2055 averaged across 40 members from CCSM3 over East Asia. Solid lines and dashed lines indicate positive values and negative values with contour levels of 0.2hPa (51 yr)^-1^ (a-b) and 2gpm (51 yr)^-1^ (c-d), respectively. Stippling and thick contours indicate the trends are statistically robust (i.e., at least 90% of 40 members produce the same sign trends).

**Fig 2:**
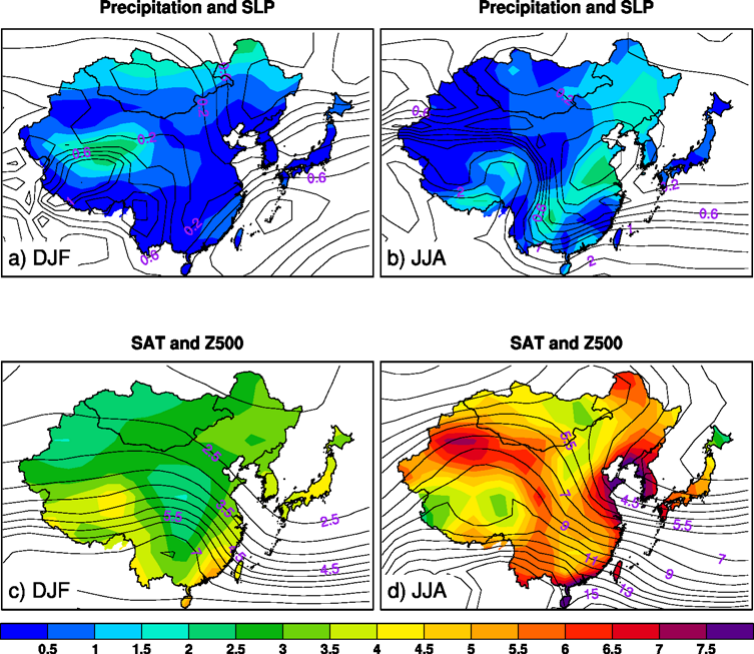
Signal to noise ratio maps for precipitation (color) and sea level pressure (contour), and surface air temperature (color) and 500hPa geopotential height (contour) in boreal winter (DJF) and summer (JJA) during 2005–2055 over East Asia.

Here, we focus on the period of 2005–2055 for each member over East Asia. We consider four important climate parameters: precipitation, near-surface air temperature (SAT), sea level pressure (SLP) and 500hPa geopotential height (Z500) in both boreal winter (December-February) and summer (June-August), and estimate their linear least-square trends over this 51 years period. Given that much of precipitation changes are affected by internal variability (Figs 1 and 2), we use k-means cluster analysis [18] to detect the occurrence of internal variability-induced precipitation trend patterns over East Asia. Generally, the k-means cluster analysis is good to relate local-scale precipitation changes with the atmospheric circulation patterns [19]. We then examine the distribution of SAT, SLP and Z500 trends according to the k-means precipitation type members. Note that the k-means clustering via the Hartigan and Wong AS-136 algorithm is performed (the source code is available at http://www.ncl.ucar.edu/Document/Functions/Built-in/kmeans_as136.shtml [20]). The model projection data used in this study is available at https://www.earthsystemgrid.org/dataset/ucar.cgd.ccsm.output.html.

To capture an optimal number of k-means nodes, a series of different nodes are performed. We find that four nodes are good enough to obtain the dominant modes of projected precipitation trends and also sufficiently small to hinder unduly similar k-means patterns. Based on domain-wide pattern correlation between internally-generated precipitation trends and different k-means nodes (Fig A in S1 File), internally-generated precipitation trends over East Asian domain are categorized into four nodes (Fig B in S1 File). To test sensitivity of internally-generated precipitation trend patterns to the numbers of k-means nodes, we also examine 2-node (Fig C in—S1 File), 8-node (Fig D in—S1 File) and 16-node (Fig E in S1 File) k-means cluster in the boreal winter. We conclude that a 2-node k-means cluster is too few to capture the diversity of internally-generated precipitation trend patterns, while 8-node and 16-node k-means cluster generates too many similar precipitation patterns. These results suggest that the 4-node k-means cluster is the best. Similar k-means node-count analyses for the boreal summer also confirm this.

## Results

### The Relative Role of the External Radiative Forcing and the Internal Variability

If assuming the global climate model would be perfect, by averaging across the 40 member ensembles, the stochastic sequences of unpredictable, internally-generated variability contained in each model realization can be reduced to reveal the response of the climate system to time-varying external radiative forcing. The externally-forced response represented by the 40 member ensemble mean can then be subtracted from individual ensemble run to extract the contribution of internal variability. The relative importance of the internally-generated and externally-forced components of climate trends over East Asia is investigated in this section.

The boreal winter and summer precipitation and SAT trends produced by the 40 member ensemble mean over East Asia, along with corresponding changes in atmospheric circulation trends (SLP and Z500), are displayed in Fig 1. The externally-forced wintertime precipitation trends show positive values across northern East Asia but negative values in southern China, southern Korea Peninsula and southern Japan (Fig 1A). However, statistically robust precipitation trends (i.e., more than 90% of the 40 members produce the same sign trends), in response to the external radiative forcing, are seen only over a small part of western and northeastern China and northern Mongolia. The non-robust precipitation trends over the major areas in East Asia reflect the important impact of internal climate variability. In boreal summer, the ensemble mean precipitation changes show wide wet trends across much of East Asia except some limited areas of western and southeastern China where dry trends occur (Fig 1B). Statistically robust wet trends mainly occur along the northeast-southwest oriented zone in the eastern China. And statistically robust dry trends are visible only in the southeastern tip of China. In general, the results indicate that the projected precipitation changes over most areas of East Asia in both winter and summer seasons are not statistically robust owing to internal climate variability.

In contrast, statistically robust widespread warming across East Asia appears in both winter and summer seasons (Fig 1C and 1D). For example, in winter, nearly homogeneous warming (1–3°C/51 yr) occupy almost the entire domain except some small areas where stronger (3–4°C/51 yr) or weaker (<1°C/51 yr) warming trends appear (Fig 1C). Similarly, the ensemble mean SAT in summer show widely robust strong warming trends (3–5°C/51 yr) across the whole East Asia, with the strongest warming (>6°C/51 yr) appearing in the northwestern East Asia (Fig 1D). The wide robust warming over East Asia in both winter and summer reflects the robust climate response to the increasing GHGs emissions in future. Consistent with the increased SAT, the atmospheric geopotential heights in 500hPa pressure levels also display positive trends over the whole East Asia. The positive 500hPa geopotential height trends are strong and widely robust across the 40 ensembles, compared to sea level pressure trends (Fig 1A and 1B). It is interesting that the precipitation changes appear to be more dynamically consistent with the sea level pressure changes, particularly in summer when the northeastern Asia climate is dominated by monsoonal circulations. For instance, the ensemble mean sea level pressure trends show statistically robust anti-cyclonic trends along the southeastern China coast and less statistically robust cyclonic trends in the eastern China. This may explain the dry trends in the southeastern China and the wet trends in the eastern China (recall Fig 1B).

To assess the relative impacts of internal variability and external forcing on future climate trends, we calculate the signal-to-noise ratio (SNR), which is defined as the absolute values of the ensemble mean precipitation (SAT, SLP and Z500) trends divided by the standard deviation of the differences between the individual member precipitation (SAT, SLP and Z500) trends and the 40 member ensemble mean (i.e., ensemble spread) at each model grid point. If the signal-to-noise ratio value is less than 1, the impact of internal climate variability exceeds that of the external forcing on the projected climate trends (i.e., the uncertainty is large).

The SNR maps of the projected precipitation, SAT, SLP and Z500 are shown in Fig 2. The SNRs of the projected precipitation in both seasons are low with values being less than 1 over most parts of East Asia except some small areas of the interior China, Korea Peninsula and northern Mongolia where the SNR values are slightly higher (1–2.5) (Fig 2A and 2B). The spatial distributions of the SNRs are similar to those of the robustness of the externally-forced precipitation trend patterns in both seasons (recall Fig 1A and 1B). Namely, the areas with the higher-than-one SNRs generally correspond to the areas with statistically robust externally-forced precipitation trends. In contrast to the low SNRs of precipitation trends, high SNRs of the wintertime SAT trends occur in many parts of East Asia with values of 2–3 over northern and central China, Mongolia and higher values of 3–5 in the other regions of East Asia (Fig 2C). Moreover, SNRs of summer SAT trends generally exhibit larger values in excess of 5 over most areas of East Asia except small parts in the southwestern and northern China and northeastern Japan where SNR values are 2.5–4 (Fig 2D). These results reveal that internally-induced uncertainty in summer projected SAT trends is considerably lower than that in winter over the major parts of East Asia. This is probably because winter hemisphere climate, compared to summer hemisphere climate, is more influenced by internal variability such as atmospheric synoptic weather systems and tropical climate signals, etc.

The much lower SNRs of the projected precipitation trends compared to those of the SAT trends in both seasons imply that the projected changes in precipitation are more impacted by internal climate variability. This is probably because precipitation, compared to SAT, is more affected by complicated atmospheric internal processes (e.g., weather and frontal systems, convection and cloud formation with strong nonlinearity, moisture transports, internal waves due to local orographic forcing, etc.). The trends in SLP and Z500 in both seasons show generally increasing SNRs from the north to the south (Fig 2), which suggests that the tropical atmospheric circulations are less influenced by internal variability. Additionally, the SLP trends are much more affected by internal climate variability compared to the Z500 trends. The relatively high uncertainties of the SLP—that can be viewed as a rough representative of lower-level atmospheric circulation except the Tibetan Plateau area—may contribute to the large uncertainties of projected precipitation trends. The results are consistent with previous studies [2, 4], which have argued that there is generally less confidence in projections of precipitation trends than those of SAT trends at regional scales.

### Internally-Generated Atmospheric Circulation Trend Patterns that Affect Future Precipitation and SAT Trends

Our results shown above suggest that precipitation trends in the both seasons suffer from large uncertainties owing to internal climate variability that appear to be related to uncertainties in the lower-level atmosphere circulations. We thus apply k-means cluster analysis to depict the occurrence of highly generalized internally-induced precipitation trend patterns across the 40 members. To facilitate the generalized precipitation trends classification, we employ four nodes over the East Asian domain that best represent the internally-induced patterns. We then group the internally-induced SAT, SLP, and Z500 trend patterns by composing each ensemble member that belongs to the corresponding k-means cluster. We evaluate the possible influences of the internally-induced changes in atmospheric circulation patterns on the uncertainties of the projected precipitation and SAT trends.

**Fig 3:**
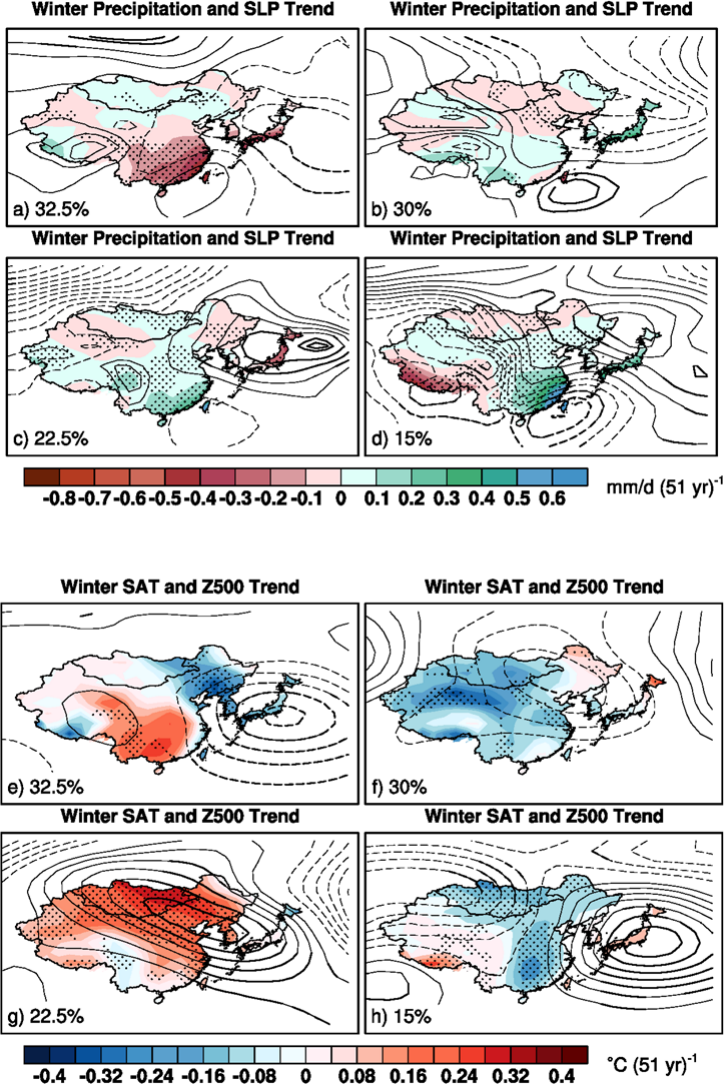
K-means cluster-derived internal variability-induced trend maps of winter precipitation (color) and sea level pressure (contour), and SAT (color) and 500hPa geopotential height trends (contour) with contour levels of 0.15hPa (51 yr)^-1^ (a-d), 2gpm (51 yr)^-1^ (e-h), respectively. Solid lines and dashed lines indicate positive values and negative values, with the zero contours being omitted. Stippling and thick contours indicate where at least 67% of each type members agree on the sign of trends. Percentage on the bottom corner of each map depicts the pattern frequency of occurrence.

The occurrences of wintertime internally-generated precipitation, SAT and atmospheric circulation trends are categorized in Fig 3. The most frequent pattern (32.5% of total occurrence) shows cyclonic circulation (i.e., negative pressure) trends at both the sea level and 500hPa level in the western North Pacific and East Asia (Fig 3A and 3E). This cyclonic circulation trends help bring dry air from inland into the area along the southeastern China to southern Japan and may contribute to the dry trends over these regions. The northern part of the cyclonic circulation trends helps transport warm air from the ocean westward into the northeastern East Asia, partly explaining the weak wet trends and cold SAT trends there. The dry trends over southern China co-occur with warm SAT trends there. The second most frequent internally-induced pattern (30% of total occurrence) shows cyclonic circulation trends over East Asia (Fig 3B and 3F), especially at 500hPa pressure level. The wide cooling trends across major areas of East Asia is well corresponding to the cyclonic circulation trends. The wet trends in Japan and South Korea also correspond closely to the trough of the cyclonic circulation trends. The third most frequent internally-induced pattern (22.5% of total occurrence) display strong anti-cyclonic circulation (i.e., positive pressure) trends over the entire East Asia at 500hPa level with a localized anti-cyclonic SLP trend pattern centered around Japan (Fig 3C and 3G). Correspondingly, wide warming across East Asia and dry trends around Japan appear. Besides, the southern part of the anti-cyclonic circulation trends at 500hPa level helps advect warm and wet air from the western Pacific Ocean westward into the southern China, generating wet trends there. Finally, the least frequent internally-generated pattern (15% of total occurrence) show anti-cyclonic circulation trends at 500hPa level over the western North Pacific in the vicinity of the Japan Islands and cyclonic circulation trends along the southeastern China coast (Fig 3H and 3D). This helps transport warm and wet airs from the western North Pacific westward into the interior of the eastern Asia, producing wet and cooling trends there.

Those internal atmospheric circulation trend patterns over East Asia are linked with broad northern hemisphere wintertime circulation trend patterns. The most frequent pattern (32.5%; Figs Fa and Ga in S1 File) is reminiscent of a negative phase of the Arctic Oscillation-like (AO-like) trend pattern [21], with positive pressure trends in the polar area and negative pressure trends in mid-latitudes. The second frequent pattern (30%; Figs Fb and Gb in S1 File) is characterized by a wavy structure with a wave number of three in the mid-high latitudes, similar to a negative phase of the east-based North Atlantic Oscillation-like (NAO-like) trend pattern [22]. The third frequent pattern (22.5%; Figs Fc and Gc in S1 File) resembles the positive phase of the Pacific-North American-like (PNA-like) trend pattern [23]. The least frequent pattern (15%; Figs Fd and Gd in S1 File) bears relevance to a negative phase of the west-based NAO-like trend pattern in the Atlantic sector and Western Pacific trend pattern in the North Pacific [22]. These results suggest that the internal atmospheric circulation trends over East Asia are closely linked with the semi-hemispheric atmospheric circulation trends.

**Fig 4:**
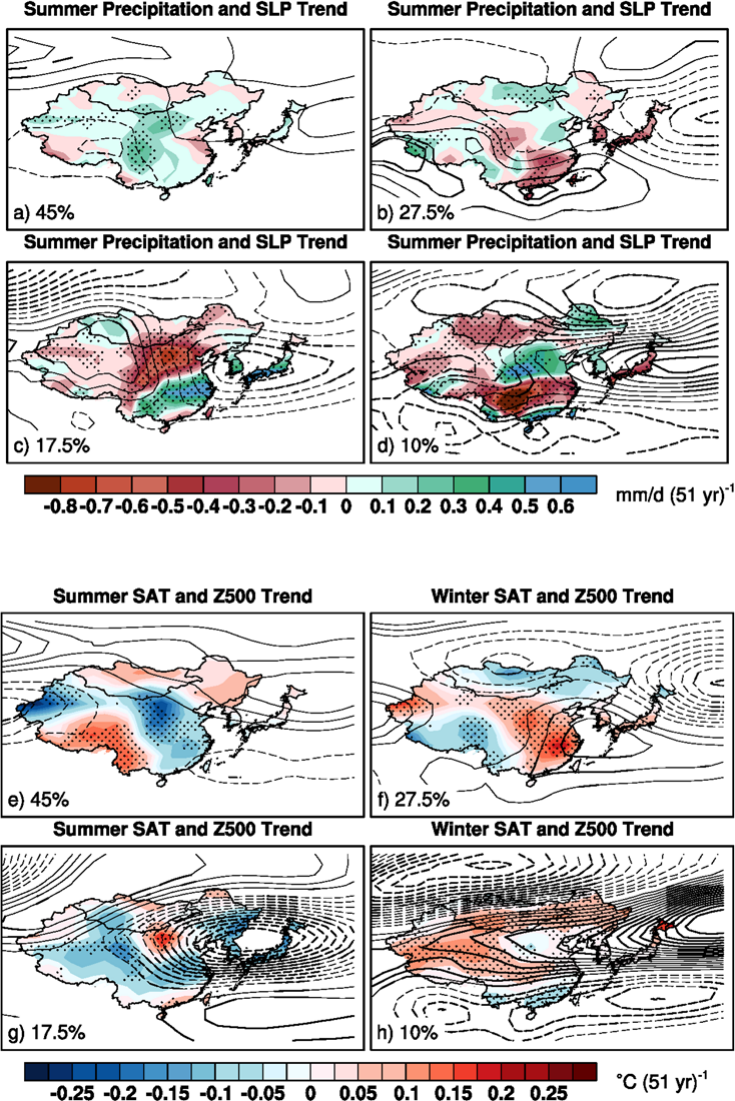
As in Fig 3, but for boreal summer. Note that contour intervals are 0.15hPa (51 yr)^-1^ and 1m (51 yr)^-1^_,_ respectively.

Similar to those in the winter, internally-generated atmospheric circulation trends in boreal summer can also affect the projected precipitation and SAT trends. The most frequent pattern (45%) characterizes quasi-barotropic anti-cyclonic circulation trends across northern East Asia and cyclonic circulation trends over southern China (Fig 4A and 4E). This North-South dipole trend pattern helps transport moist maritime airs from the western Pacific into China that accounts for the wet and cooling trends in the central and eastern China. The anti-cyclonic circulation trends in the northern pole also help induce warm trends over northeastern East Asia. The second frequent pattern (27.5% of 40 members) shows cyclonic circulation trends east of Japan and anti-cyclonic circulation trends along the southeastern China coast (Fig 4B and 4F). Correspondingly, dry and warm trends appear in the Eastern China, Korea Peninsula and Japan. This pattern is partly similar to an opposite polarity of the first frequent pattern. In the third frequent pattern (17.5% of 40 members), while anti-cyclonic circulation trends dominate in the western China, strong barotropic cyclonic circulation trends occupy the eastern China, Korea Peninsula and Japan (Fig 4C and 4G). Accordingly, a zonally elongated band of wet trends forms over the southeastern China, Korea Peninsula and Japan, and dry trends occur in the north of eastern China, which is associated with a southward shifted Meiyu-Baiyu rainfall front. The trends at 500hPa levels show a tripole pattern in the eastern Asia with two anti-cyclonic circulations straddling the cyclonic circulation along the eastern China, Korea Peninsula and Japan. This is reminiscent of the Pacific-Japan-like trend pattern [24]. The least frequent pattern (10% of 40 members) also shows a tripole pattern but with an opposite polarity in general (Fig 4H). Consistently, the precipitation and SAT trend patterns also display an opposite polarity with dry trends along the southeastern China, Korea Peninsula and Japan.

Our results suggest that the magnitudes and spatial patterns of the projected summer precipitation and SAT trends, like those in winter, are also impacted by the internally-generated large-scale atmospheric circulation trends. These internally-generated atmospheric circulation trends in East Asia are also linked with broad structures in the northern hemisphere. For instance, the most frequent pattern (45%) is related to a weak annular pattern in the mid-high latitudes, reminiscent of a positive phase of the AO-like trend pattern (Figs Ha and Ia in S1 File). The second frequent pattern (27.5%) is related to a wavy structure with a western Pacific-like trend pattern over East Asia (Figs Hb and Ib in S1 File). The third and fourth frequent patterns (17.5% and 10% of 40 members) display an opposite polarity of a strong annular pattern with a dipole structure between the polar region and mid-latitudes (Figs Hc-d and Ic-d in S1 File) except in East Asia where the tripole structure resembles the Pacific-Japan pattern [24]. Note that the dry (wet) trends often co-occur with the hot (cool) trends in East Asia, suggesting that changes in atmospheric circulations play an important role in driving these internally-generated precipitation and SAT trends.

## Discussions

Our arguments may be potentially subject to some caveats. It is generally assumed that the externally-forced response and internally-generated variability of the climate system are linearly cumulative, whether or not they may non-linearly interact with each other remains to be an open question. Recent studies have demonstrated that climate models also have some irreducible errors in simulating the atmospheric internal variability [25–27] and climate changes in response to external radiative forcing. This emphasizes the need for an explicitly probabilistic, risk-based perspective to evaluate the relative roles of external forcing and internal variability in projected regional climate changes. Large ensembles of numerical integrations produced by a single model [10] and/or multi-models are essential to better estimate uncertainty in regional climate change and climate risk management due to internal variability of the climate system and/or model errors. Moreover, attention should also be paid to the role of the ocean’s internal variability, which can modulate large-scale atmospheric circulation patterns and remotely affect regional climate trends. Concerning the non-negligible systematic biases in modelling internal variabilities, new observations with high accuracy are essential to improve the estimate of internal climate variabilities.

## Conclusions

Internally-induced uncertainties in the projected East Asian precipitation and SAT trends in both boreal winter and summer during 2005–2055 are examined on the basis of CCSM3 40 member ensembles of climate change simulations. To yield the 40 member ensembles of climate change projections, the CCSM3 model is run with the same timely-varying radiative forcing scenario (SRES A1B) but initiated from a suite of different atmospheric initial states. We present compelling evidence to show that the internally-induced uncertainty in the projected SAT trends in winter is generally larger than that in summer. Moreover, compared to SAT, projection of precipitation trends in both seasons is subject to much larger uncertainty owing to internal climate variability. This suggests that there is generally less confidence in projected changes in precipitation than those in SAT, as was illustrated by [3, 4]. Besides, the sea level pressure shows considerable uncertainties, which is in stark contrast to the much less uncertainties of the mid-level atmospheric circulation (500hPa). Based on k-means cluster analysis, we identify four distinct patterns of internal variability-induced precipitation and SAT trends in the both seasons over East Asia. We find that a substantial portion of the projected internally-induced precipitation and SAT trends could be, at least to a certain extent, attributable to changes in internal large-scale atmospheric circulation patterns. Our results confirm that changes in internal atmospheric variability are an important source of uncertainty in future regional climate change projections, as elucidated by [28]. Thus, much caution is required when assessing regional climate change projections, given the important impacts of internal climate variability.

## Supporting Information

S1 File The Supporting Information file includes supplementary figures: Fig A-Fig I. (PDF)
