# Supplementary material for: Internal Variability-Generated Uncertainty in East Asian Climate Projections Estimated with 40 CCSM3 Ensembles
Source: PLoS One. 2016 Mar 1;11(3):e0149968. doi: 10.1371/journal.pone.0149968 (PMC4773150; doi:10.1371/journal.pone.0149968)
Supplement: S1 File — (PDF) [file pone.0149968.s001.pdf]

Supporting Information for

Internal variability-generated uncertainty in East Asian climate projections estimated with 40 CCSM3 ensembles

Shuai-Lei Yao[1,2], Jing-Jia Luo[3], Gang Huang[1,4,*]

[1]*State Key Laboratory of Numerical Modeling for Atmospheric Sciences and Geophysical Fluid Dynamics, Institute of Atmospheric Physics, Chinese Academy of Sciences, Beijing 100029, China*

[2]*University of Chinese Academy of Sciences, Beijing 100049, China*

[3]*Bureau of Meteorology, Melbourne, VIC 3008, Australia*

[4]*Joint Center for Global Change Studies, Beijing 100875, China*

Re-submitted to *PLOS ONE, Feb. 20, 2016*

* Correspondence to:

Dr. Gang Huang, P. O. Box 9804, Beijing 100029, China. E-mail: hg@mail.iap.ac.cn.

Tel: +86-10-82995312. Fax: +86-10-82995150

This document contains Supplementary Figures referenced in the main text.

Supplementary Figures

Fig. A Sensitivity of pattern similarity to number of k-means cluster nodes for boreal winter (DJF, a) and summer (JJA, b). To capture an adequate number of k-means nodes, we calculate the max/min pattern correlations of 40 member internal variability-induced precipitation trends with their matching k-means cluster node pattern (blue/green pluses) and the maximum/minimum k-means pattern to k-means pattern correlation (magenta/purple asterisks) for a suite of different k-means node counts (3, 4, 5, 6, 7 and 8) in DJF and JJA over East Asian domain. We also compute the mean pattern correlation of unforced precipitation trends and their matching k-means node patterns (red crosses). We aim to select an optimum number of k-means cluster nodes so that: 1) the minimum pattern correlation of 40 member internal variability-induced precipitation trends is relatively large; 2) the maximum k-means pattern to k-means pattern correlation is relatively small; and 3) the mean pattern correlation of 40 member internal variability-induced precipitation trends is relatively large. We identify that four k-means nodes are typically adequate to capture projected internal variability-induced precipitation trend patterns and the number is small enough to depict the dominant precipitation trend patterns over East Asia.

Fig. B 4-node k-means cluster analyses of 40 member internal variability-induced precipitation trends in boreal winter over East Asia. The values in the bottom corner denote the k-means node and the pattern frequency of occurrence, respectively.

Fig. C 2-node k-means cluster analyses of 40 member internal variability-induced precipitation trends in boreal winter over East Asia. The values in the bottom corner denote the k-means node and the pattern frequency of occurrence, respectively.

Fig. D 8-node k-means cluster analyses of 40 member internal variability-induced precipitation trends in boreal winter over East Asia. The values in the bottom corner denote the k-means node and the pattern frequency of occurrence, respectively.

Fig. E 16-node k-means cluster analyses of 40 member internal variability precipitation trends in boreal winter over East Asia. The values in the bottom corner denote the k-means node and the pattern frequency of occurrence, respectively.

Fig. F The northern hemisphere k-means cluster-derived internal variability-induced trend maps of winter precipitation (color) and sea level pressure (contour) with a contour level of 0.4hPa $(51\ yr)^{-1}$ (a-d). Solid lines and dashed lines indicate positive values and negative values, with the zero contours being omitted. Stippling and thick contours indicate where at least 67% of each type members agree on the sign of trends. Percentage on the bottom corner of each map depicts the pattern frequency of occurrence.

Fig. G The northern hemisphere k-means cluster-derived internal variability-induced

trend maps of winter surface air temperature (color) and 500hPa geopotential height (contour) with a contour level of 4gpm (51 yr)$^{-1}$ (a-d). Solid lines and dashed lines indicate positive values and negative values, with the zero contours being omitted. Stippling and thick contours indicate where at least 67% of each type members agree on the sign of trends. Percentage on the bottom corner of each map depicts the pattern frequency of occurrence.

Fig. H The northern hemisphere k-means cluster-derived internal variability-induced trend maps of summer precipitation (color) and sea level pressure (contour) with a contour level of 0.4hPa (51 yr)$^{-1}$ (a-d). Solid lines and dashed lines indicate positive values and negative values, with the zero contours being omitted. Stippling and thick contours indicate where at least 67% of each type members agree on the sign of trends. Percentage on the bottom corner of each map depicts the pattern frequency of occurrence.

Fig. I The northern hemisphere k-means cluster-derived internal variability-induced trend maps of summer surface air temperature (color) and 500hPa geopotential height (contour) with a contour level of 2gpm (51 yr)$^{-1}$ (a-d). Solid lines and dashed lines indicate positive values and negative values, with the zero contours being omitted. Stippling and thick contours indicate where at least 67% of each type members agree on the sign of trends. Percentage on the bottom corner of each map depicts the pattern frequency of occurrence.

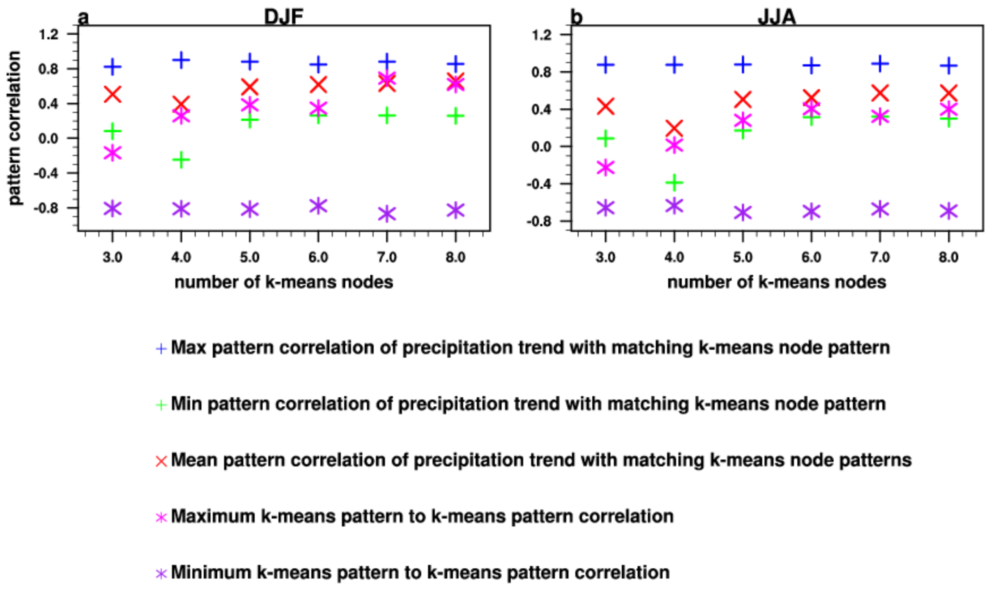

Fig. A Sensitivity of pattern similarity to number of k-means cluster nodes for boreal winter (DJF, a) and summer (JJA, b). To capture an adequate number of k-means nodes, we calculate the max/min pattern correlations of 40 member internal variability-induced precipitation trends with their matching k-means cluster node pattern (blue/green pluses) and the maximum/minimum k-means pattern to k-means

pattern correlation (magenta/purple asterisks) for a suite of different k-means node counts (3, 4, 5, 6, 7 and 8) in DJF and JJA over East Asian domain. We also compute the mean pattern correlation of unforced precipitation trends and their matching k-means node patterns (red crosses). We aim to select an optimum number of k-means cluster nodes so that: 1) the minimum pattern correlation of 40 member internal variability-induced precipitation trends is relatively large; 2) the maximum k-means pattern to k-means pattern correlation is relatively small; and 3) the mean pattern correlation of 40 member internal variability-induced precipitation trends is relatively large. We identify that four k-means nodes are typically adequate to capture projected internal variability-induced precipitation trend patterns and the number is small enough to depict the dominant precipitation trend patterns over East Asia.

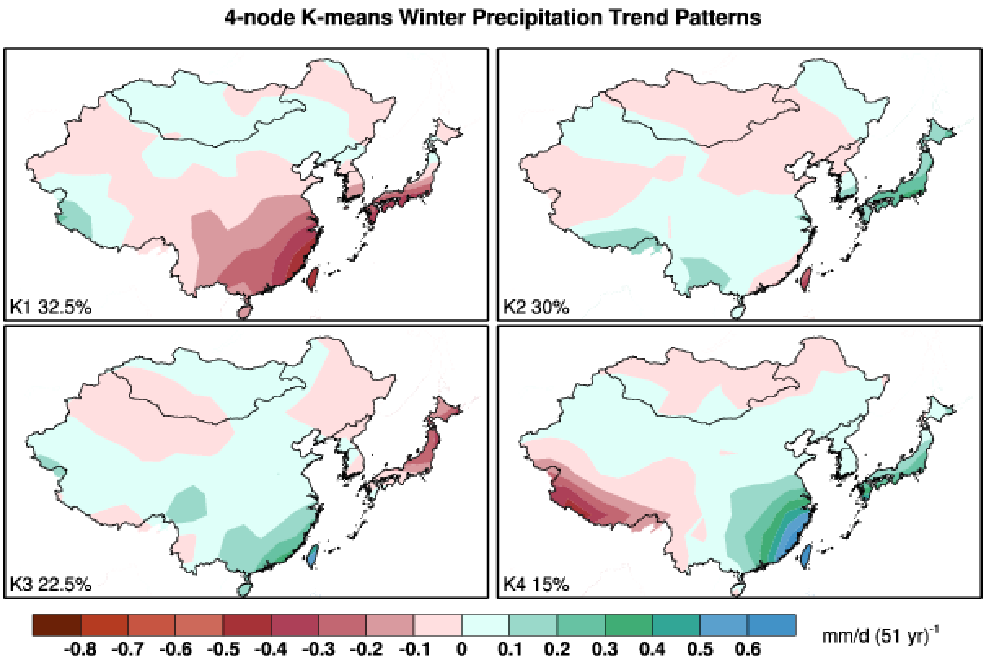

Fig. B 4-node k-means cluster analyses of 40 member internal variability-induced precipitation trends in boreal winter over East Asia. The values in the bottom corner denote the k-means node and the pattern frequency of occurrence, respectively.

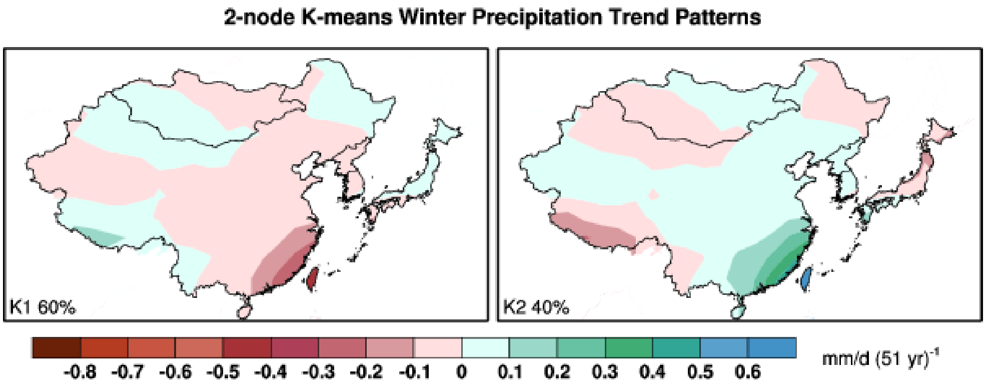

Fig. C 2-node k-means cluster analyses of 40 member internal variability-induced precipitation trends in boreal winter over East Asia. The values in the bottom corner denote the k-means node and the pattern frequency of occurrence, respectively.

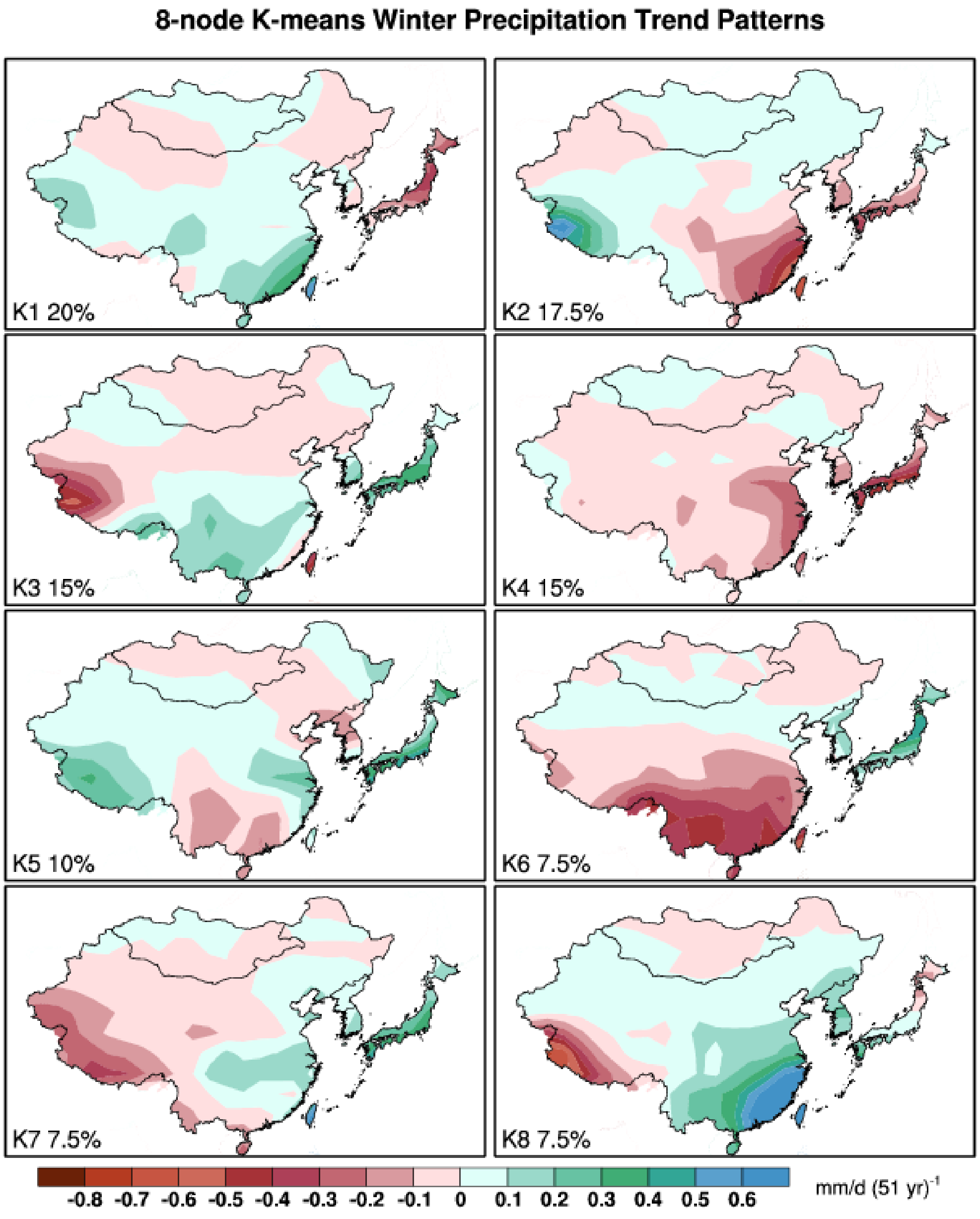

Fig. D 8-node k-means cluster analyses of 40 member internal variability-induced precipitation trends in boreal winter over East Asia. The values in the bottom corner denote the k-means node and the pattern frequency of occurrence, respectively.

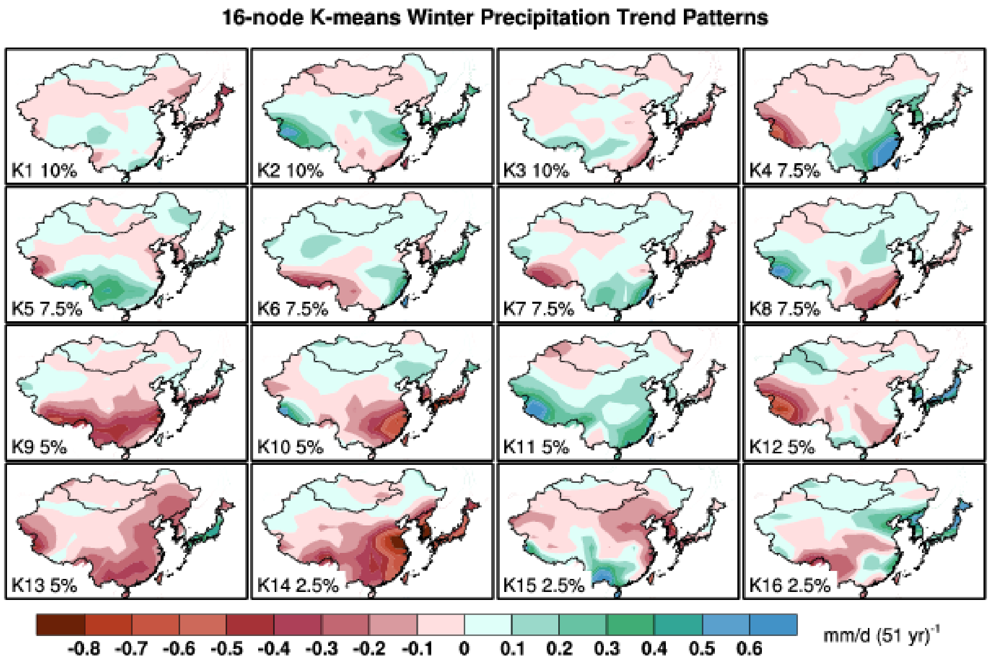

Fig. E 16-node k-means cluster analyses of 40 member internal variability precipitation trends in boreal winter over East Asia. The values in the bottom corner denote the k-means node and the pattern frequency of occurrence, respectively.

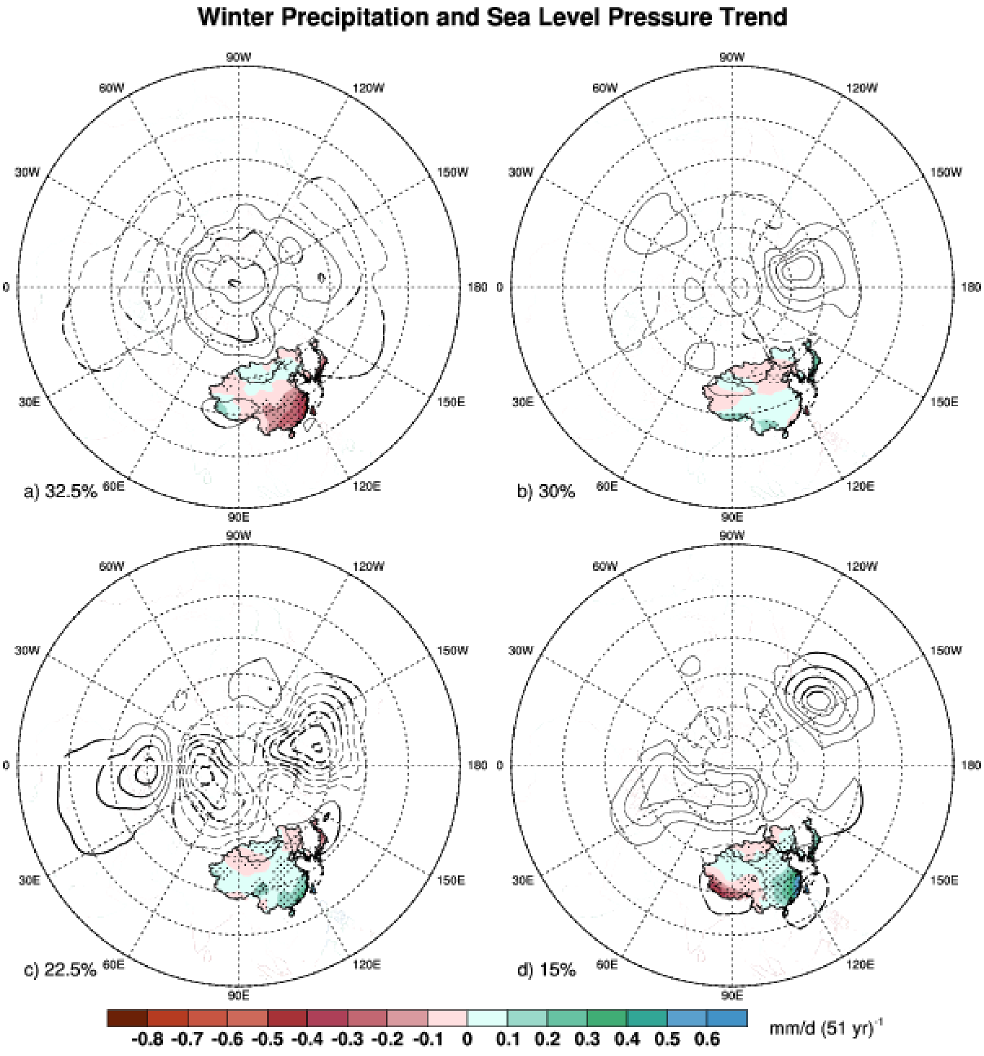

Fig. F The northern hemisphere k-means cluster-derived internal variability-induced trend maps of winter precipitation (color) and sea level pressure (contour) with a contour level of 0.4hPa (51 yr)$^{-1}$ (a-d). Solid lines and dashed lines indicate positive values and negative values, with the zero contours being omitted. Stippling and thick contours indicate where at least 67% of each type members agree on the sign of trends.

Percentage on the bottom corner of each map depicts the pattern frequency of occurrence.

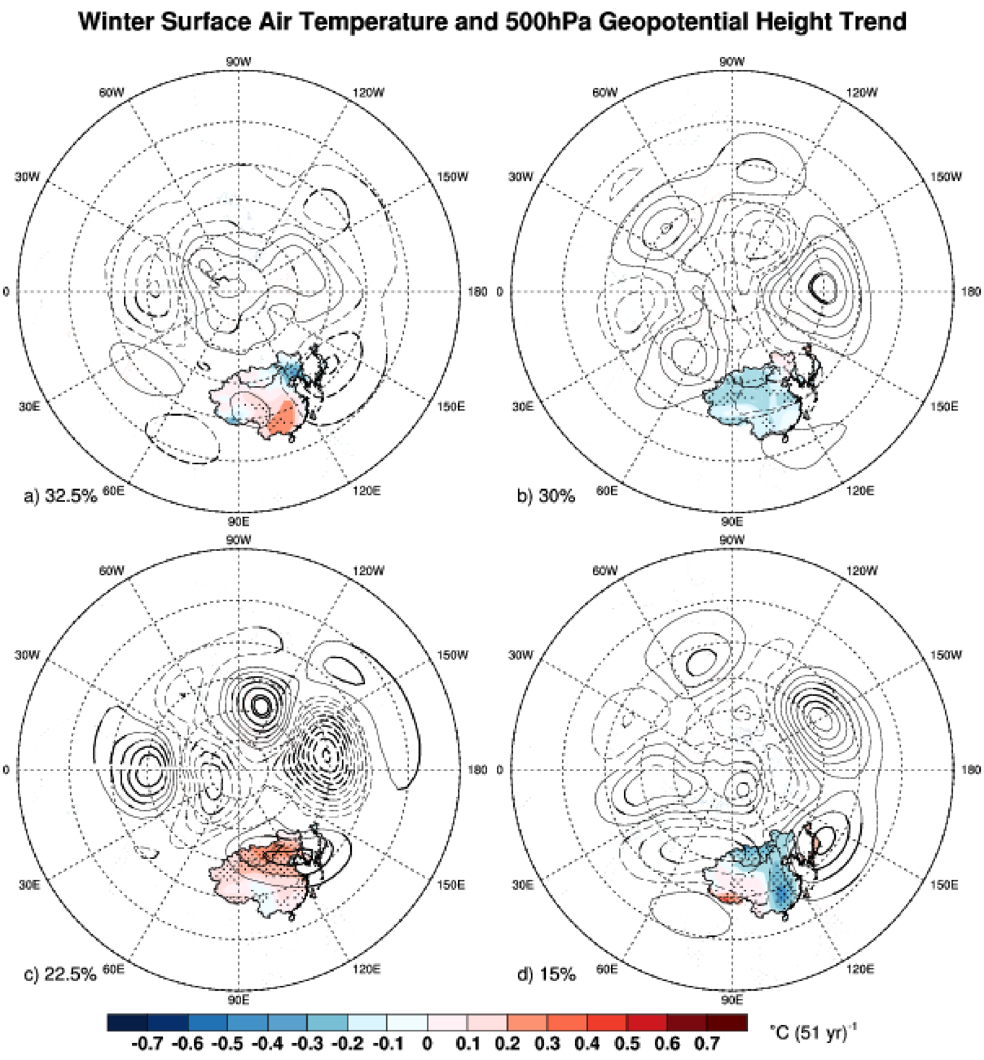

Fig. G The northern hemisphere k-means cluster-derived internal variability-induced trend maps of winter surface air temperature (color) and 500hPa geopotential height (contour) with a contour level of 4gpm (51 yr)$^{-1}$ (a-d). Solid lines and dashed lines indicate positive values and negative values, with the zero contours being omitted. Stippling and thick contours indicate where at least 67% of each type members agree

on the sign of trends. Percentage on the bottom corner of each map depicts the pattern frequency of occurrence.

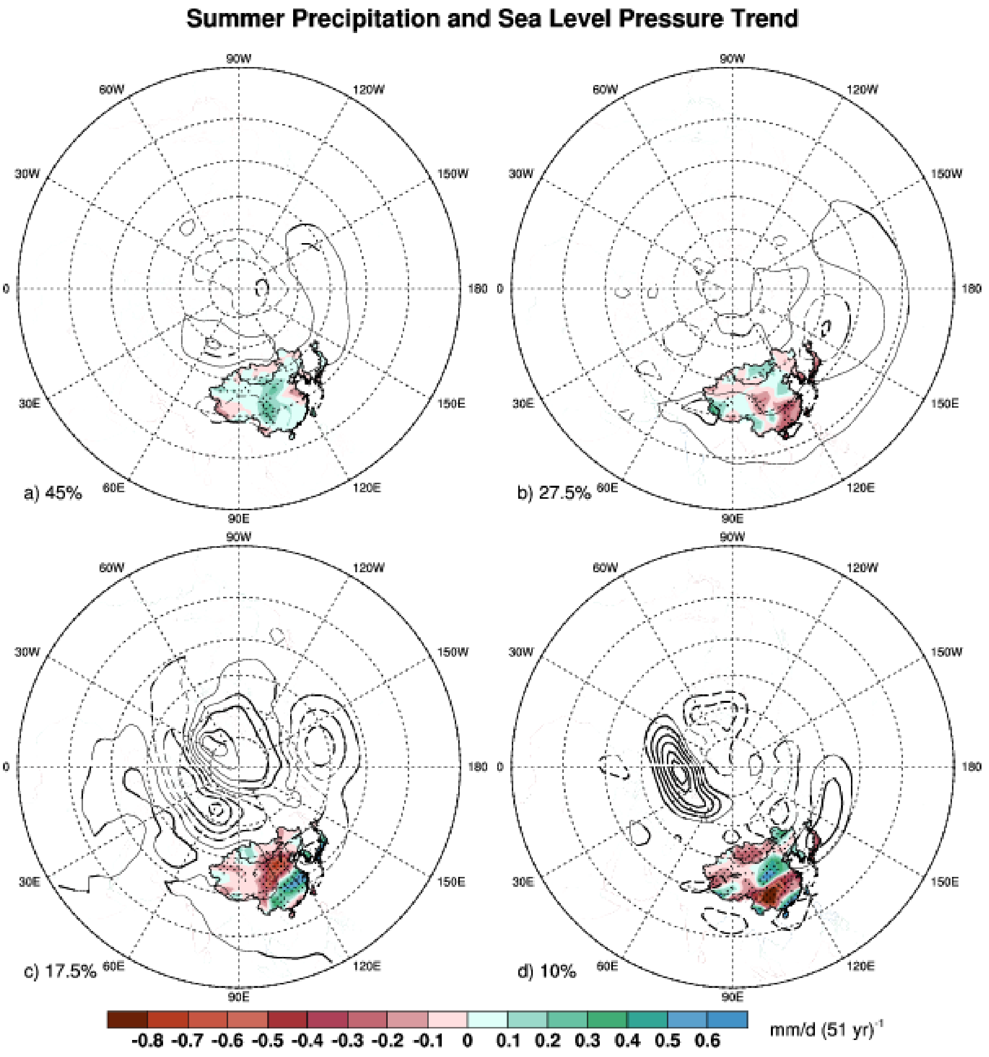

Fig. H The northern hemisphere k-means cluster-derived internal variability-induced trend maps of summer precipitation (color) and sea level pressure (contour) with a contour level of 0.4hPa (51 yr)$^{-1}$ (a-d). Solid lines and dashed lines indicate positive values and negative values, with the zero contours being omitted. Stippling and thick contours indicate where at least 67% of each type members agree on the sign of trends.

Percentage on the bottom corner of each map depicts the pattern frequency of occurrence.

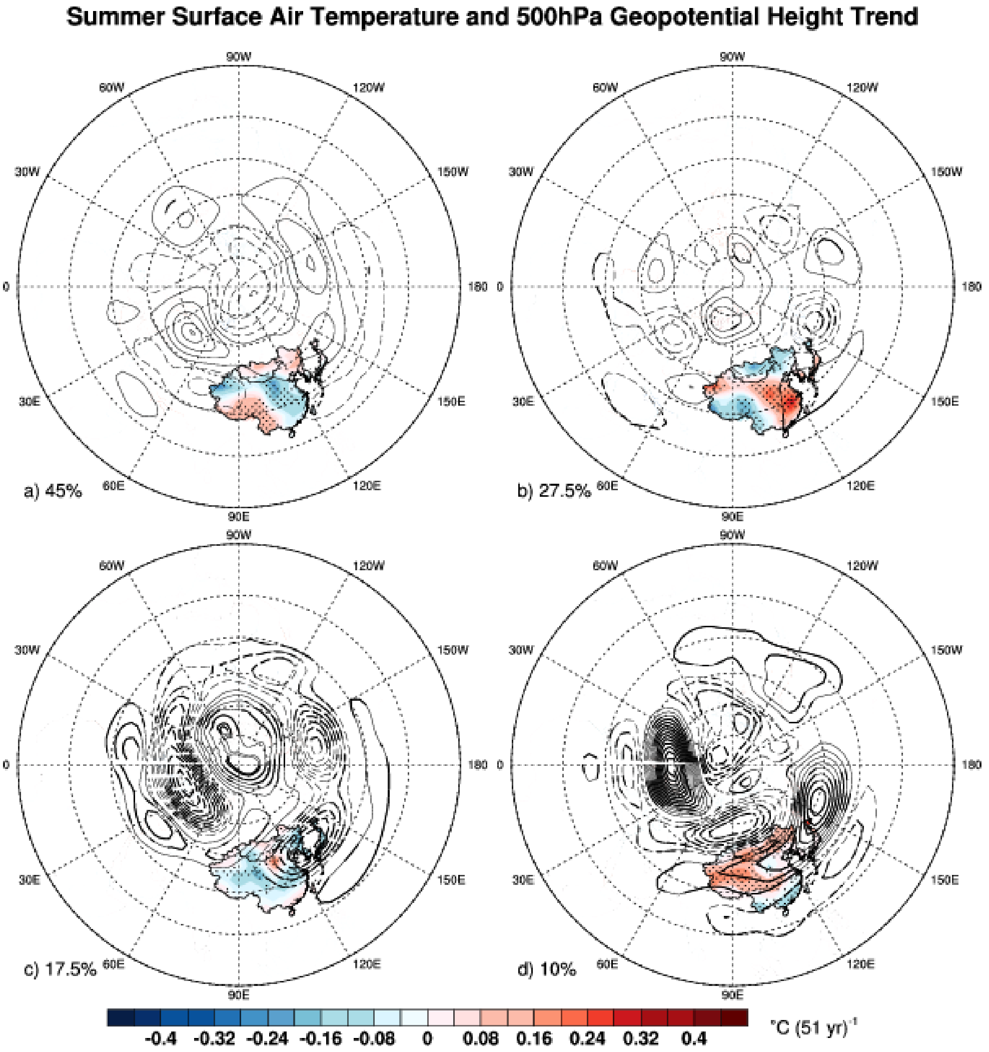

Fig. I The northern hemisphere k-means cluster-derived internal variability-induced trend maps of summer surface air temperature (color) and 500hPa geopotential height (contour) with a contour level of 2gpm (51 yr)$^{-1}$ (a-d). Solid lines and dashed lines indicate positive values and negative values, with the zero contours being omitted. Stippling and thick contours indicate where at least 67% of each type members agree

on the sign of trends. Percentage on the bottom corner of each map depicts the pattern frequency of occurrence.
